# Flow-Based *In Situ* Synthesis of Covalent Organic Framework Thin Films and Liquid-Phase Quartz Crystal Microbalance with Dissipation Monitoring (QCM-D) Analysis of Adsorption Kinetics

**DOI:** 10.1021/acsami.6c06434

**Published:** 2026-06-01

**Authors:** Wen-Yi Yu, Pei-Chen Huang, Yun-Wen You, I-Chia Huang, Cheng-Si Tsao, Jing-Jong Shyue

**Affiliations:** † Department of Materials Science and Engineering, 33561 National Taiwan University, Taipei 10617, Taiwan; ‡ Research Center for Applied Sciences, 12381 Academia Sinica, Taipei 11529, Taiwan; § Program in Semiconductor Devices, Materials, and Hetero-integration, Graduate School of Advanced Technology, National Taiwan University, Taipei 10617, Taiwan; ∥ National Synchrotron Radiation Research Center, Hsinchu 30076, Taiwan

**Keywords:** covalent organic framework, quartz crystal microbalance, flow-based synthesis, film growth, adsorption kinetics, host–guest interactions

## Abstract

Covalent organic frameworks (COFs) constitute a class of functional porous materials with high stability and tunable porosity, rendering them attractive for adsorption-based applications. To study the interaction between COFs and target molecules, the quartz crystal microbalance with dissipation monitoring (QCM-D), which offers high sensitivity to mass changes with a sampling rate of ∼50 ms, is a well-suited tool for analyzing adsorption processes in real time. However, current QCM-D-based adsorption studies have remained largely qualitative, primarily relying on comparison of the magnitudes of frequency shifts. This occurs because the intrinsic mass of the film coated onto QCM-D sensors is often not determined, which prevents the quantification of the adsorption capacity. To address this challenge, a flow-based strategy to directly grow homogeneous COF thin films on sensor surfaces under QCM-D monitoring was established. This approach facilitates reliable mass determination, robust film attachment, and reproducible film preparation with precise control over film growth. To account for the contribution of the embedded solvent during film deposition, deuterated solvent exchange was performed. Through the use of methylene blue as a model adsorbate, liquid-phase QCM-D measurements revealed adsorption capacities of ∼31 mg·g^–1^ for triformylphloroglucinol-phenylenediamine (TpPa) COF and ∼259 mg·g^–1^ for TpPa-SO_3_H within 60 min. It was found that a single kinetic model cannot describe the entire process. Instead, the pseudo-second-order (PSO) model best described the initial surface-controlled phase, whereas subsequent uptake was dominated by intraparticle diffusion (IPD). A hybrid kinetic model with a time-weighted function successfully captured the transition between these two stages and provided insights into the difference in mass transportation in TpPa and TpPa-SO_3_H. This methodology provides a platform for high-speed, quantitative and real-time liquid-phase analysis of COF–guest interactions with high potential for extension to other porous materials.

## Graphical abstract

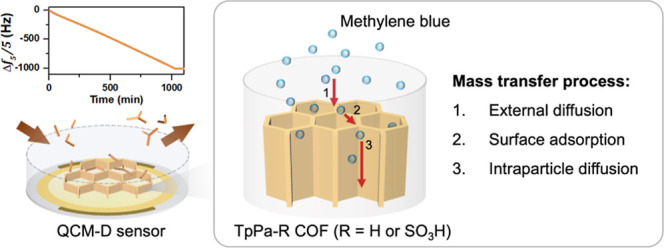

## Introduction

Covalent organic frameworks (COFs) constitute a class of crystalline, porous materials synthesized from lightweight, nonmetal elements interconnected through robust covalent bonds. Since the first reports of boroxine-linked COF-1 and boronate ester-linked COF-5 by the Yaghi group in 2005,^1^ COFs have rapidly evolved because of their structural tunability,^2^ large surface area,^3^ and exceptional thermal and chemical stability.^4^ These features render them promising for diverse applications, such as wastewater treatment,^5–7^ drug delivery,^8^ gas storage and separation,^9–11^ catalysis,^12^ and chemical detection.^13,14^ To advance these applications, particularly those involving interactions between the COF host and guest molecules, a precise and real-time analytical platform is needed to evaluate the adsorption performance and study interaction kinetics in detail. The quartz crystal microbalance with dissipation monitoring (QCM-D), which is based on the converse piezoelectric effect, is an ideal tool for this purpose. The QCM-D offers extremely high mass sensitivity at the level of nanograms per square centimeter (ng·cm^–2^) and provides real-time data with a temporal resolution <0.1 s. These features enable the direct monitoring of adsorption processes in both gas and liquid environments and provide insights into the underlying mechanisms across various fields.^15–18^

Compared with commonly employed batch measurements, QCM-D enables continuous operando monitoring without uncertainties introduced by intermittent sampling and separation procedures of adsorbent and adsorbate-containing environments during measurements. In addition, the constant supply of fresh solution maintains a constant adsorbate concentration, which aligns with the fundamental assumption in common adsorption kinetic models.^19^ Furthermore, beyond the surface adsorption process, the unidirectional diffusion of the guest from the outermost liquid–solid interface into the film interior along the thickness direction facilitates a more distinct observation of the intraparticle diffusion. However, despite the effectiveness of the QCM-D for real-time operando analysis, its data quality critically depends on the preparation of stable, homogeneous, adherent, rigid, and sufficient surface coverage of thin films on the sensing surface,^20^ particularly in liquid-phase analyses.

Several strategies have been developed to deposit COFs on QCM sensors, such as drop casting,^21–24^ spray coating,^25^ direct growth,^26,27^ and flow-based methods.^28–31^ Each method exhibits distinct advantages and limitations. Drop casting is the most widely employed approach, in which presynthesized COF powders are packed as films during the drying process. Although simple, this method often requires repeated deposition cycles to achieve uniformity, resulting in thick films that exceed the penetration depth of the acoustic wave in liquid-phase QCM-D measurements (∼250 nm in water for 5 kHz crystals, and the range decreases with increasing overtone)^32^ and suffer from cracks or low adhesion. Spray coating, in which COF colloidal suspensions are employed as the ink in combination with air-brush techniques, offers a more rapid method to fabricate large-area films. However, controlling the film thickness and homogeneity remains challenging. Direct growth offers a bottom-up route to enhance adhesion by immersing self-assembled monolayer (SAM)-modified QCM crystals with designed surface functional groups into precursor solutions, facilitating surface chemical reactions and *in situ* COF growth on the surface. However, the complexity of COF self-assembly kinetics is often neglected. Two equations are typically employed to describe the competing reactions in the bulk solution and at the liquid–substrate interface^30^

1 $$r_{bulk}=k_{bulk}{\left[monomer\right]}^{2}$$

2 $$r_{surface}=k_{surface}\left[monomer\right]$$

where *r* is the reaction rate, and *k* is the reaction constant. The bulk-phase reaction (eq 1) generally follows pseudo-second-order (PSO) kinetics, whereas the surface reaction at the liquid–substrate interface (eq 2), where the number of active sites on the surface is constant and limited, follows pseudo-first-order (PFO) kinetics. In practice, the bulk-phase reaction is dominant because of the relatively high monomer concentration for the direct growth method, yielding rapid and stochastic nucleation. In addition, the degree of freedom of the orientation of surface-confined functional groups is lower than that of free molecules in solution. Hence, the reaction rate at the surface is suppressed. As a result, the films produced via direct growth often exhibit nonuniform thickness and rough surfaces.^30,31^

Moreover, a major challenge across these approaches is the uncertainty in quantifying the deposited COF mass, making it difficult to derive the adsorption capacity, which is a key absorbent performance index in terms of the adsorbed mass per unit adsorbent mass (g·g^–1^), from the QCM-D data. To overcome this limitation, flow-based *in situ* synthesis has emerged as a promising alternative. By continuously delivering reactant solutions with a low and steady monomer concentration through a confined flow module, the flow-based method aims to minimize rapid nucleation in the premixed solution of reactants and to restrict the reaction to the solution–substrate interface. In this strategy, the reactants diffuse across the boundary layer and react at the substrate, facilitating controlled bottom-up growth of homogeneous and crack-free COF films. Simultaneously, the COF particles formed in the solution are removed under continuous flow conditions, further eliminating the contamination of film surfaces. Coupling this flow strategy with *in situ* QCM-D monitoring provides real-time tracking of COF growth, thereby ensuring precise control over the film mass and enabling direct quantification of the deposited film.

On the basis of these features, flow-based synthesis of direct‑growth β-ketoenamine-linked COF films with and without a –SO_3_H pendent group within the pores on SAM-modified QCM-D sensors are reported herein. In the continuous flow approach, the supply and mixing of reactants are regulated in space and time by precisely controlling the flow rate of the precursor solutions^33,34^ to produce uniform, adherent, and sufficiently thin films for further QCM-D studies of adsorption kinetics. Furthermore, to determine the intrinsic mass of the as-grown films, a deuterated solvent-exchange experiment was conducted to determine the contribution of the solvent confined within the porous framework during *in situ* synthesis, thereby providing a reliable basis for calculating the weight of the empty COF and its adsorption capacity. While most QCM-based COF adsorption studies to date have focused on gaseous cargos, molecular cargos in the liquid phase have been far less explored. To address this gap, aqueous methylene blue (MB) solutions were selected as modeling guest cargos, and their adsorption kinetics were examined. By introducing a time-weighted function and multiple kinetic models, the dominant mechanisms were identified across different stages of the adsorption process. The effect of functional groups on the kinetics was then examined. Overall, this combination of flow-based synthesis and QCM-D monitoring provides a versatile platform for advancing studies of the adsorption kinetics of molecules by COFs that can be extended to a wide range of porous materials to provide greater insights into adsorption mechanisms and capacities, especially in the liquid phase.

## Experimental Section

### Materials

1,3,5-Triformylphloroglucinol (Tp, 98 wt %), 1,4-phenylenediamine (Pa, 98 wt %), and 1,4-phenylenediamine-2-sulfonic acid (Pa-SO_3_H, 98 wt %) were obtained from TCI. 6-Amino-1-hexanethiol hydrochloride, dimethyl sulfoxide (DMSO, 99.5%), dimethyl sulfoxide-d6 (DMSO-*d*_6_, 99.9 at. % D), deuterium oxide (D_2_O, 99.9 at. % D), and an MB solution (0.05 wt % in water) were purchased from Sigma-Aldrich. Absolute ethanol (99.8%) was purchased from Honeywell. Ethanol-*d*_6_ (EtOH-*d*_6_, 95% in D_2_O) was obtained from Combi-Blocks. Dioxane (98%) was purchased from J.T. Baker. Acetone (99%) and mesitylene (98%) were obtained from Thermo Scientific. Glacial acetic acid (99.7%) was acquired from SHOWA. All the chemicals were used as received without further purification.

### Quartz Crystal Microbalance with Dissipation Measurements

QCM-D experiments were performed using a Q-Sense E4 system (Biolin Scientific, Sweden) to continuously monitor the growth process of COF films, determine the encapsulated solvent content, and evaluate the adsorption kinetics of MB into COF films. 5 MHz AT-cut quartz crystal sensors with gold electrodes on both sides (QSX301, Biolin Scientific, Sweden) were employed. The frequency shift ( $\frac{{\Delta f}_{n}}{n}$ ) and the change in energy dissipation (Δ*D*_*n*_) were recorded for the fundamental resonance frequency (*n* = 1) and six overtones (*n* = 3, 5, 7, 9, and 13). For clarity, data from the fifth overtone (*n* = 5, i.e., 25 MHz) are used in more detailed analysis of kinetics.

For rigid, thin, and uniform films where Δ*D*_*n*_ is negligible or minimal, the Sauerbrey model was applied to relate the frequency shift (Hz) to the adsorbed mass (ng·cm^–2^)^35^

3 $$\Delta m=-C\frac{\Delta f_{n}}{n}$$

where *C* is the mass sensitivity constant (17.7 ng·cm^–2^·Hz^–1^ for 5 MHz sensors).

### Fabrication of TpPa-R COF-Coated QCM-D Sensors

COF-coated QCM-D sensors were prepared based on the methodology previously reported by the Börjesson group.^28–30^ Gold-coated QCM-D sensors were first cleaned with a standard ammonia-peroxide mixture (APM, NH_4_OH/H_2_O_2_/Milli-Q water, v/v = 1:1:5) at 85 °C for 30 min. Thereafter, the sensors were sonicated twice in Milli-Q water, dried under a nitrogen stream, and treated by a UV/ozone cleaner (maker) for 60 min. To achieve surface functionalization with a SAM, the cleaned sensors were immersed in a 1 mM ethanolic solution of 6-aminohexane-1-thiol (3 mL) containing 1.2 M HCl (0.5 mL) and kept in the dark for 24 h.^36^ The amino-terminated QCM sensors with Au–S anchoring groups were then rinsed and sonicated in absolute ethanol for 3 min, dried under a nitrogen stream, and positioned within the QCM-D flow module (QFM 401; Biolin Scientific, Sweden).

Two model COFs, i.e., TpPa-R (R = H or SO_3_H), were synthesized in this work. For simplicity, TpPa-H is referred to as TpPa in the following discussion. After the module was assembled with the SAM-coated QCM sensor, flow-based synthesis was initiated. For TpPa synthesis, the module was first flushed with absolute EtOH as the background solvent and stabilized at 55 °C until a steady-state QCM-D signal was established. Solutions of the aldehyde building block Tp (0.2 mM in EtOH) and the amine building block Pa (0.2 mM in EtOH) were subsequently introduced. To synthesize TpPa-SO_3_H, the module was flushed with an EtOH/DMSO mixture (19:1 v/v) and stabilized at 50 °C before the introduction of the Tp solution (0.2 mM in EtOH) and the Pa-SO_3_H solution (0.2 mM in EtOH/DMSO = 9:1 v/v). In both synthesis processes, the precursor solutions were delivered by two independently controlled syringe pumps at a constant flow rate of 13 μL·min^–1^. After the volume above the sensor was replaced by the reactant solution, the shifts in the resonance frequency ( $\frac{{\Delta f}_{n}}{n}$ ) and change in energy dissipation (Δ*D*_*n*_) were continuously recorded. Film growth continued until the target frequency shift was attained, as measured via operando QCM-D analysis, after which the module was flushed with the background solvent again for 60 min. The resulting TpPa-R COF-coated sensors were dried under nitrogen and stored under vacuum.

### Synthesis of TpPa and TpPa-SO_3_H Bulk Powders

TpPa and TpPa-SO_3_H powders were synthesized following the procedure reported by Kandambeth et al.^37^ Specifically, COFs were obtained via Schiff-base reactions between Tp (31.5 mg, 0.15 mmol) and the corresponding amine monomers, i.e., Pa (24 mg, 0.225 mmol) or Pa-SO_3_H (42.3 mg, 0.225 mmol). The reactions were conducted in 3 mL of a 1:1 v/v mesitylene/dioxane mixture, with 0.25 mL of 3 M acetic acid serving as the catalyst. The mixture was sealed in a glass vial and heated at 115 °C for 24 h. The resulting solids were collected and purified via sonication in dioxane three times and then in acetone three times, followed by activation under vacuum at 75 °C for 8 h and subsequent storage under vacuum.

### Characterization of Materials

The morphology and coverage of the as-synthesized COF films were assessed via scanning electron microscopy (SEM; FEI Nova200 NanoSEM, FEI, USA) in high-vacuum secondary electron (SE) mode. Cross-sectional images were obtained with a beam energy of 5 kV (spot size: 3.5) and a tilt angle of −10°. The film thickness and root-mean-square (RMS) roughness were determined via atomic force microscopy (AFM; Veeco Innova SPM, Bruker, USA) in tapping mode with aluminum-coated cantilevers (force constant: ∼5 N/m; resonance frequency: ∼150 kHz). The elemental composition was analyzed through X-ray photoelectron spectroscopy (XPS; PHI 5000 VersaProbe, ULVAC-PHI, Japan). Measurements were performed using Al Kα X-rays at a power of 25 W and a beam size of 100 μm. The energy analyzer was set with a constant pass energy of 117.4 eV, and the photoelectron takeoff angle was set to 45°. A homemade goniometer was used to measure the water contact angle and the image was analyzed using software provided by Dataphysics (SCA20, German). COF linkage formation was verified by Fourier transform infrared spectroscopy (FTIR; VERTEX 70, Bruker, USA). The precursor powder was prepared as a KBr plate (0.5 wt %) and measured in transmission mode. In addition, the thin COF film was characterized directly on the substrate using reflection mode. The zeta potential was measured with a 90Plus Zetasizer (Brookhaven Instruments Corp., USA), in which the phase analysis light scattering (PALS) technique is employed. The COF powder sample was prepared as a solution (0.125 mg·mL^–1^) in a 1 mM NaCl aqueous electrolyte. The pH was adjusted via an automated titrator (Titrator T70, Mettler-Toledo, USA) using 0.1 M HCl and NaOH. Analyses were conducted at 25 °C using a 35 mW red laser (660 nm). A voltage of 5 V was applied across the AQ-600 electrodes with a field reversal frequency of 2 Hz. Data were collected over 10 repetitions of 30 cycles, and the Smoluchowski model was employed to determine the zeta potential from electrophoretic mobility.

Crystalline structures were investigated using synchrotron-based grazing-incidence wide-angle X-ray scattering (GIWAXS) at the National Synchrotron Radiation Research Center (NSCCR, Taiwan). GIWAXS measurements were conducted with a synchrotron X-ray source (λ = 1.02738 Å; *E* ≈ 12 keV) at an incident angle of 0.5°, with diffraction signals collected by a 2D MarCCD SX165 detector (Rayonix, USA). Data were converted into Cu Kα_1_ equivalent patterns in GSAS-II software (Argonne National Laboratory, USA). Powder X-ray diffraction (PXRD; Rigaku TTRAX3, Rigaku, Japan) was also employed to obtain the diffraction pattern of the bulk powder sample. PXRD patterns were collected using Cu Kα_1_ radiation (λ = 1.54056 Å) over a 2-theta range of 5°–30° (diffraction angle) in steps of 0.02° with 2theta/omega scan mode. Simulated diffraction patterns were generated from structural models reported in the literature.^37,38^

### Solvent Fraction Determination via Deuterated Solvent Exchange

Since the COF films were deposited from liquid media, they remained solvated as synthesized. Thus, the measured frequency shift ${\left(\frac{{\Delta f}_{n}}{n}\right)}_{measured}$ included contributions from both the intrinsic framework mass ${\left(\frac{{\Delta f}_{n}}{n}\right)}_{film\;mass}$ and the solvent retained in the pores ${\left(\frac{{\Delta f}_{n}}{n}\right)}_{solvent}$

4 $${\left(\frac{{\Delta f}_{n}}{n}\right)}_{measured}={\left(\frac{{\Delta f}_{n}}{n}\right)}_{film\;mass}+{\left(\frac{{\Delta f}_{n}}{n}\right)}_{solvent}$$

To quantify the solvent content within the as synthesized COF, a deuterated solvent exchange experiment was conducted using the background solvent and its deuterated analog.^16,39–41^ For the TpPa film, EtOH served as the background solvent, whereas an EtOH/DMSO mixture (19:1 v/v) was employed for the TpPa-SO_3_H film. Each background solvent was first flushed until equilibrium, defined as the baseline (Δ*f* = 0, Δ*D* = 0). The corresponding deuterated solvent (EtOH-*d*6 or an EtOH-*d*6/DMSO-*d*_6_ mixture) was then introduced for 60 min before switching back to the solvent with a natural isotope ratio. To ensure more rapid and complete solvent exchange, these experiments were performed using thinner COF films with a measured frequency shift of approximately −200 Hz during film deposition. Because of the density difference between the solvent (ρ_solvent_: 0.789 g·cm^–3^ for EtOH; 0.805 g·cm^–3^ for the EtOH/DMSO mixture) and its deuterated form (ρ_*d*‑solvent_: 0.892 g·cm^–3^ for EtOH; 0.907 g·cm^–3^ for the EtOH/DMSO mixture), the mass of the same volume of solvent encapsulated in the film differed, resulting in a frequency shift. From this shift, the contributions of the solvent content ${\left(\frac{{\Delta f}_{n}}{n}\right)}_{solvent}$ and the solvent fraction *s*_solvent_ were calculated as follows^39^

5 $${\left(\frac{{\Delta f}_{n}}{n}\right)}_{solvent}=\frac{{\left(\frac{{\Delta f}_{n}}{n}\right)}_{COF,Sol.}-{\left(\frac{{\Delta f}_{n}}{n}\right)}_{blank,Sol.}}{\left(\frac{\rho_{d-solvent}}{\rho_{solvent}}\right)-1}$$

6 $$s_{solvent}=\frac{{\left(\frac{{\Delta f}_{n}}{n}\right)}_{solvent}}{{\left(\frac{{\Delta f}_{n}}{n}\right)}_{measured}}$$

where ${\left(\frac{{\Delta f}_{n}}{n}\right)}_{COF,Sol.}$ and ${\left(\frac{{\Delta f}_{n}}{n}\right)}_{blank,Sol.}$ are the frequency responses to the deuterated solvent for the COF-coated and unmodified (blank) sensors, respectively.

### QCM-D Analysis of the Adsorption Kinetics

Considering the difference in the wettability of different COFs, the COF-coated QCM sensors were exposed to deionized (DI) water at a flow rate of 100 μL·min^–1^ inside the flow module until steady baselines were established to ensure they were fully wetted. Aqueous MB solutions (0.01, 0.05, 0.1, and 1 mM) were then introduced at a rate of 100 μL·min^–1^ for 60 min at 25 °C. Following the adsorption experiments, the module was flushed with DI water to remove residual MB molecules. The specific adsorption capacity at a given time, *q*_*t*_ (mg·g^–1^), was determined as the ratio of the mass change due to MB uptake to the mass of the COF film. Via the use of the Sauerbrey relation (eq 3), *q*_*t*_ can be expressed as follows

7 $$q_{t}=\frac{m_{t,MB\;uptake}}{m_{film\;mass}}=\frac{{-C\left(\frac{{\Delta f}_{n}}{n}\right)}_{t,MB\;uptake}}{-C{\left(\frac{{\Delta f}_{n}}{n}\right)}_{film\;mass}}\times 10^{3}\;\frac{mg}{g}$$

where *C* is the mass sensitivity constant (17.7 ng·cm^–2^·Hz^–1^ for 5 MHz sensors), and ${\left(\frac{{\Delta f}_{n}}{n}\right)}_{t,M\;Buptake}$ and ${\left(\frac{{\Delta f}_{n}}{n}\right)}_{film\;mass}$ denote the frequency responses to MB adsorption at time *t* and the mass of the intrinsic COF film, respectively.

Adsorption kinetics were then analyzed using PFO, PSO, and intraparticle diffusion (IPD, also known as the Weber–Morris model) models with a sigmoid-like time-dependent weighting factor to determine the transition between the dominating mechanisms.

## Results and Discussion

### Preparation and Characterization of TpPa and TpPa-SO_3_H Thin Films

The continuous flow method offers distinct advantages for the controlled synthesis of COF thin films. As highlighting in the recent review,^33^ precisely regulating the flow rate allows for the modulation of the spatiotemporal profile of the monomer reaction, which ensures a consistent and homogeneous supply of reactants on the substrate. This process maintains stable local concentrations, which is essential for steady and uniform film growth. The use of low-concentration precursor solutions also reduces homogeneous nucleation in the bulk solution, thereby suppressing particle formation and contamination. Additionally, any oligomeric byproducts are swiftly removed by the solvent stream, which increases film homogeneity and purity. Moreover, these reaction conditions facilitate heterogeneous growth on the surface, thereby promoting site-specific polymerization on the substrate and yielding bottom-up formation of a continuous thin film. As shown in the schematic in Figure 1, the integration of the flow-based synthesis method with a QCM-D setup provided real-time monitoring of the growth of β-ketoenamine-linked 2D COF films (TpPa and TpPa-SO_3_H) by recording the frequency response, thereby facilitating *in situ* quantification of COF deposition.

**Figure 1:**
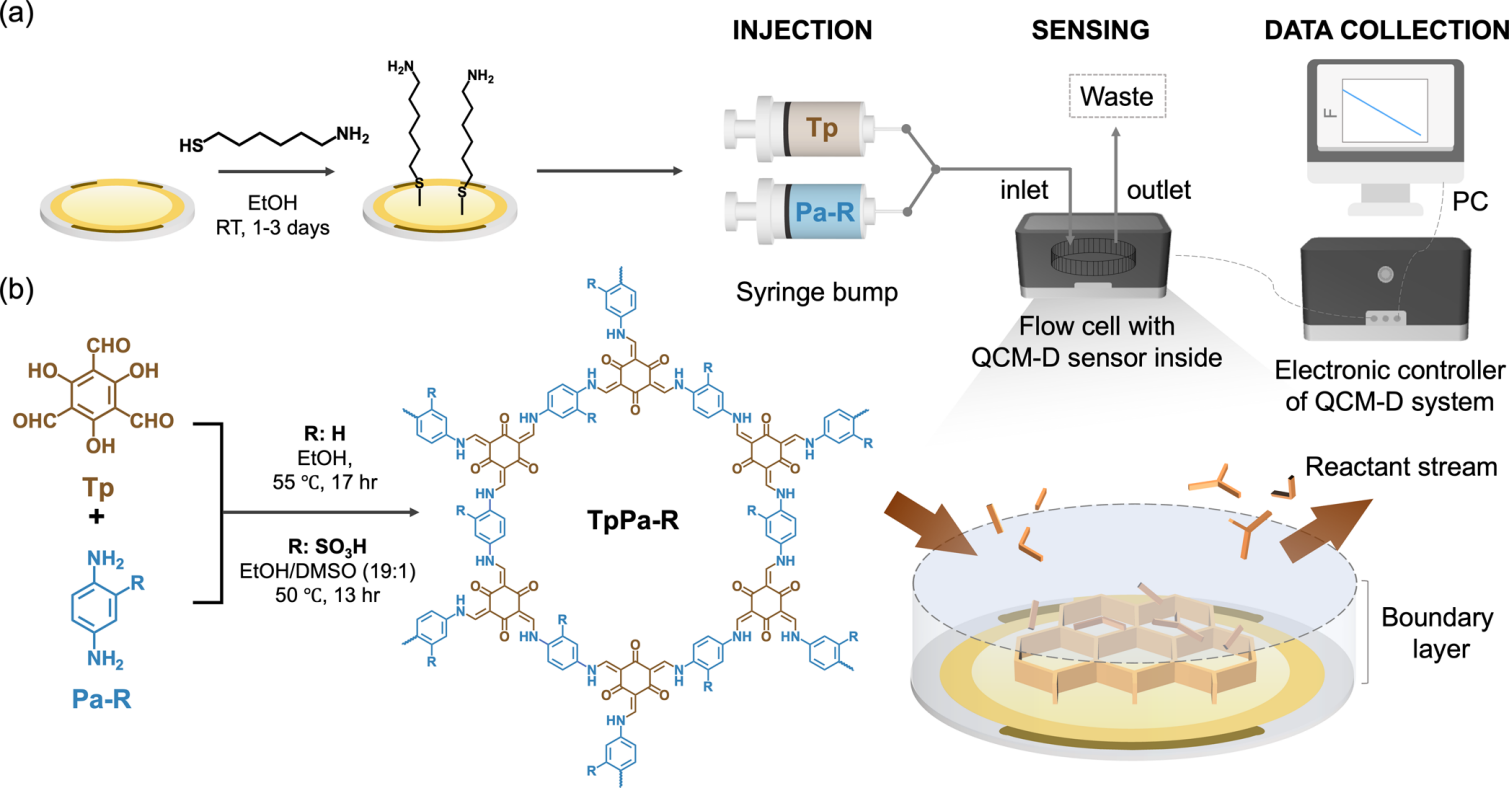
Flow-based synthesis of COF films monitored via a QCM-D based system. (a) Sketch of the flow QCM-D setup illustrating two precursor solutions being delivered by independently computer-controlled syringe pumps into a temperature-controlled flow cell containing the SAM-modified QCM-D sensor to monitor the film growth process. (b) Formation of TpPa-R (R = H or SO_3_H) through a Schiff base condensation reaction and tautomerization to produce β-ketoenamine linkage.

The frequency responses in the film growth process of TpPa and TpPa-SO_3_H are shown in Figure 2a,b, respectively. The change in the resonance frequency ( $\frac{{\Delta f}_{n}}{n}$ ) for both COFs measured at approximately 50 ms interval decreased linearly until the end of growth, indicating steady and controlled film deposition rates of −0.963 Hz·min^–1^ for TpPa and −1.23 Hz·min^–1^ for TpPa-SO_3_H (*R*_adj_^2^ = 0.999 for both COFs). Upon reintroduction of the background solvent, the frequency response remained unchanged, suggesting that the as-grown films were firmly adhered to the QCM sensor surface and that no unreacted species were freely adsorbed on the surface. Furthermore, the changes in energy dissipation (Δ*D*_*n*_) were minimal (∼1.5 × 10^–6^) across all the measured overtones (*n* = 1, 3, 5, 7, 9, and 13), thus confirming the high mechanical rigidity of the COF films. This rigidity allowed the shear wave to fully penetrate the entire film thickness, thereby justifying the use of the Sauerbrey equation (eq 3)^42^ to convert frequency shifts into mass (or thickness) changes according to a linear relationship and ensuring that all overtones effectively address the adsorption processes on the film surface.

**Figure 2:**
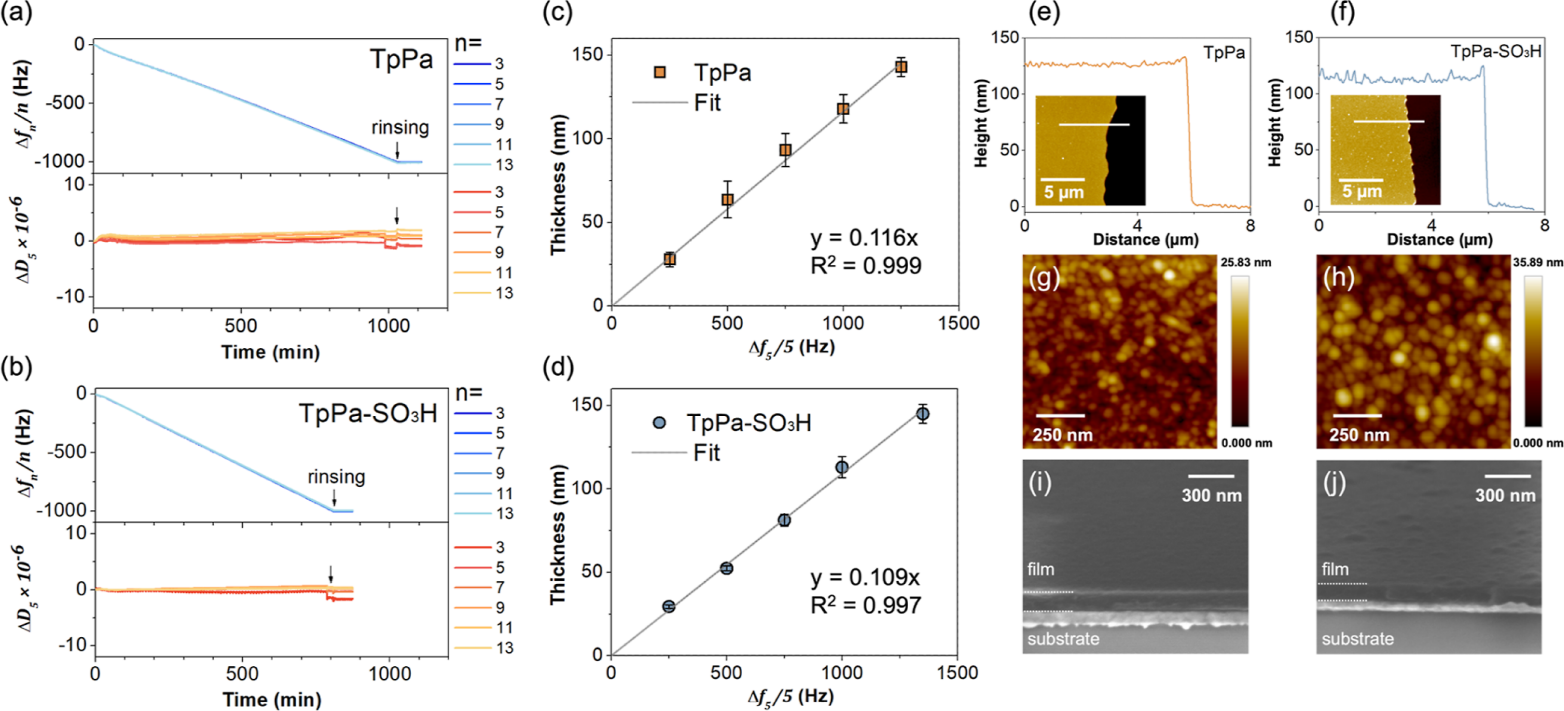
Real-time QCM-D measurement results and morphological characterization of TpPa and TpPa-SO_3_H films. (a,b) Resonance frequency shifts and energy dissipation changes during the growth of TpPa and TpPa-SO_3_H films. (c,d) Correlations between the frequency shift and film thickness for TpPa and TpPa-SO_3_H measured via AFM. (e,f) AFM height profiles of TpPa and TpPa-SO_3_H films. (g,h) Topographic AFM images of TpPa and TpPa-SO_3_H films. (i,j) Cross-sectional SEM images (−10° tilt) of TpPa and TpPa–SO_3_H films.

The linear correlation between the frequency shift ( $\frac{{\Delta f}_{5}}{5}$ , fifth overtone) and the film thickness measured via AFM is shown in Figure 2c,d. The calculated thickness-to-frequency ratios were 0.116 nm·Hz^–1^ for TpPa and 0.109 nm·Hz^–1^ for TpPa-SO_3_H, which confirmed the homogeneity of the COF and highlights the precise control of film growth at the nanometer scale. Furthermore, it is confirmed that the linear Sauerbrey model is valid for converting the frequency shift into mass, volume and thickness given the constant density and area of the deposited film. The AFM height profiles of the scratched COF films are shown in Figure 2e,f, where the thicknesses of the TpPa and TpPa-SO_3_H films are approximately 116 and 110 nm, respectively, corresponding to a frequency shift of −1000 Hz. Topographic AFM images (Figure 2g,h) revealed uniform surface topographies with low RMS roughness values of 3.75 nm for TpPa and 4.79 nm for TpPa-SO_3_H. Moreover, cross-sectional SEM images with tilt to visualize the surface (Figure 2i,j) revealed that both COFs were flat, continuous, and free of cracks, indicating complete coverage of the surface by the COF films. Although the surface roughness of the sulfonic-functionalized film was slightly greater, both films maintained excellent flatness and uniformity, rendering them ideal samples for subsequent adsorption studies using the QCM-D method.

FTIR spectroscopy was employed to examine the chemical bonding and confirm the formation of β-ketoenamine linkages. The FTIR spectra of TpPa and TpPa-SO_3_H are compared with those of their corresponding precursors in Figure 3a. The Schiff base condensation reaction was verified by the disappearance of the aldehyde C=O stretching band at 1643 cm^–1^ and the N–H stretching band within the 3100–3300 cm^–1^ range. The absence of the O—H stretching band near 3000 cm^–1^ and the C=N stretching band at approximately 1650 cm^–1^ also suggested that the initially formed enol-imine intermediates experienced irreversible tautomerization to form a more thermodynamically stable β-ketoenamine structure. The emergence of new peaks corresponding to C=C stretching at 1578 cm^–1^ and C—N stretching at 1284 cm^–1^ provided additional evidence for the successful formation of the β-ketoenamine framework.^37,43^ The bands at approximately 1028 and 1082 cm^–1^ correspond to the characteristic O=S=O symmetric and asymmetric stretching vibrations, respectively,^44^ further confirmed the incorporation of sulfonic acid groups in the TpPa-SO_3_H framework.

**Figure 3:**
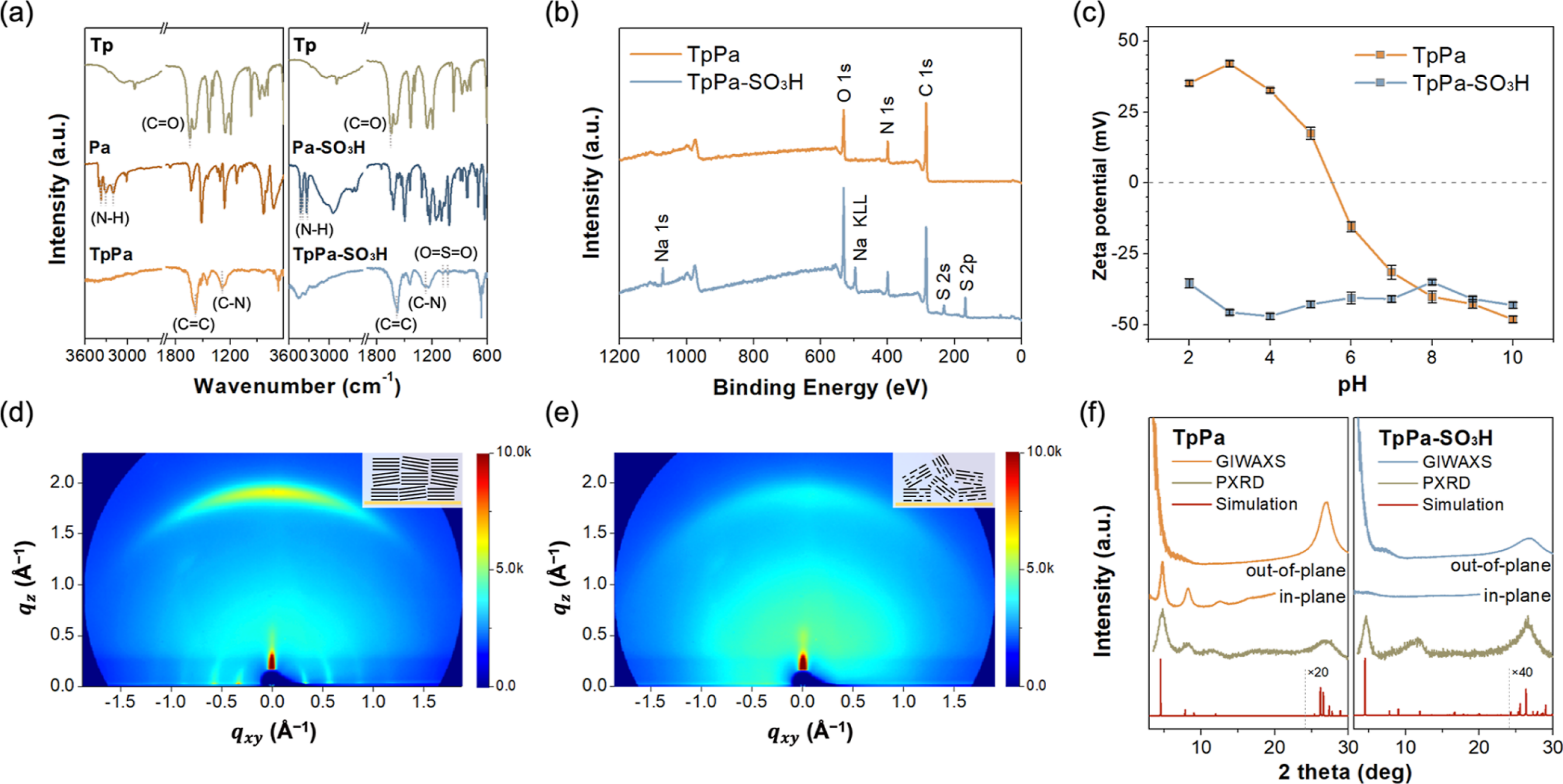
Characterization of thin film TpPa and TpPa-SO_3_H (∼110 nm) and bulk powder forms. (a) FTIR spectra of TpPa and TpPa-SO_3_H films and their respective precursors in KBr plates. (b) XPS survey spectra of TpPa and TpPa-SO_3_H films. (c) Zeta potentials of TpPa and TpPa-SO_3_H powders. (d) and (e) 2D GIWAXS images of TpPa and TpPa-SO_3_H films, respectively. (f) Comparison of the thin-film GIWAXS profiles and PXRD results of powder samples with the simulated diffraction patterns.

The XPS survey spectrum shown in Figure 3b was used to confirm the elemental composition of the COF films. For TpPa-SO_3_H, signals for C, N, O, and S were clearly observed, which is consistent with the expected chemical structure. The atomic S/N ratio of 0.51 conforms well with the theoretical stoichiometry of the TpPa-SO_3_H framework (a ratio of 0.50). A sodium signal was also identified, likely originating from airborne sodium contamination commonly present in the environment^45^ and retained as the counterion to the –SO_3_^–^ groups, with a measured Na/S ratio of 0.80. The absence of other impurity-related peaks confirmed the high purity of the synthesized films. Furthermore, the Au signal of the underlying substrate was not detected in the XPS analysis for both COF films. In conjunction with the AFM and SEM observations presented above, these results indicated that the entire surface was covered by the COF, thereby preventing direct exposure of the substrate to the surrounding media during subsequent adsorption measurements.

The zeta potentials of TpPa and TpPa-SO_3_H were measured across a pH range of 2 to 10, as shown in Figure 3c, to investigate their surface charge. With respect to TpPa, the zeta potential was positive at low pH values and gradually shifted to negative values with increasing pH, with an isoelectric point (IEP) of approximately 5.5. In contrast, TpPa-SO_3_H remained negatively charged across the entire pH range, attributable to the deprotonation of sulfonic acid (–SO_3_H) groups, which dominate the surface charge of the framework.

The 2D GIWAXS pattern of the TpPa film with arc-like diffraction spots along the in-plane *q*_*xy*_ direction (*q*_*z*_ = 0) at 0.34, 0.59, and 0.90 Å^–1^, corresponding to the (100), (110) and (210) planes, respectively, of the hexagonal lattice is shown in Figure 3d. These peaks confirmed that the hexagonal lattice of the grains aligned parallel to the substrate, with significant (100) plane reflection indicating highly ordered 1D channels and high crystallinity. Simultaneously, an out-of-plane (001) spot at *q*_*z*_ = 1.91 Å^–1^ (*q*_*xy*_ = 0) revealed π–π stacking of the ordered COF sheets along the *c* axis, which were oriented perpendicular to the substrate within approximately ±15°. The coexistence of in-plane arcs and the (001) reflection indicated that the grains were randomly rotated about the *c*-axis. The inset shows a schematic illustration of this structure. In contrast, compared with the TpPa film, the TpPa-SO_3_H film (Figure 3e) exhibited a lower crystallinity. This result can be attributed to the sulfonic acid pendent groups inside the pore disrupting the hexagonal lattice. Consequently, the in-plane *q*_*xy*_ spots were replaced by diffused diffraction rings, indicating a wide angular orientation and small grains. The decreased but remaining (001) reflection suggested reduced long-range order in-plane, but partial lamellar stacking persisted. These microscopic structural features may also contribute to the slight difference in roughness observed by AFM (Figure 2g,h).

The thin-film GIWAXS data are compared with the 1D PXRD data of the bulk powders (Figure 3f). With respect to TpPa (left panel), the positions of the diffraction spots of the thin film match those of the bulk peaks at 2θ = 4.8°, 8.2°, and 27.1° (*d* = 1.84, 1.08, and 0.33 nm, respectively), corresponding to the pore size and interlayer stacking distance. With respect to TpPa-SO_3_H, the bulk PXRD results indicate main peaks at 2θ = 4.6°, 11.6°, and 26.6° (*d* = 1.92, 0.76, and 0.34 nm, respectively), whereas the thin-film pattern reveals a distorted structure with tilted grains.

### Mass of the COF Thin Films

The determination of the mass of guest molecules adsorbed in the deposited COF films requires the quantification of solvent molecules trapped inside the pores during synthesis. Solvent exchange experiments were conducted to determine the contribution of solvents on the basis of the density difference between the solvent and its deuterated analog, which share nearly identical physicochemical properties and interactions with COFs. This approach facilitates the distinction between frequency shifts originating from the COF and from the solvent molecules retained within the pores. To ensure complete solvent exchange and a more rapid equilibrium throughout the entire film thickness, solvent exchange experiments were conducted with thinner COF films (corresponding to an as-measured frequency shift of −200 Hz during film deposition). The resulting frequency responses are shown in Figure 4a. Via the use of eqs 5 and 6, the calculated contributions of the solvent molecules inside the as-synthesized COF films were −99.5 Hz (49.7%) for TpPa and −94.0 Hz (47.0%) for TpPa-SO_3_H.

**Figure 4:**
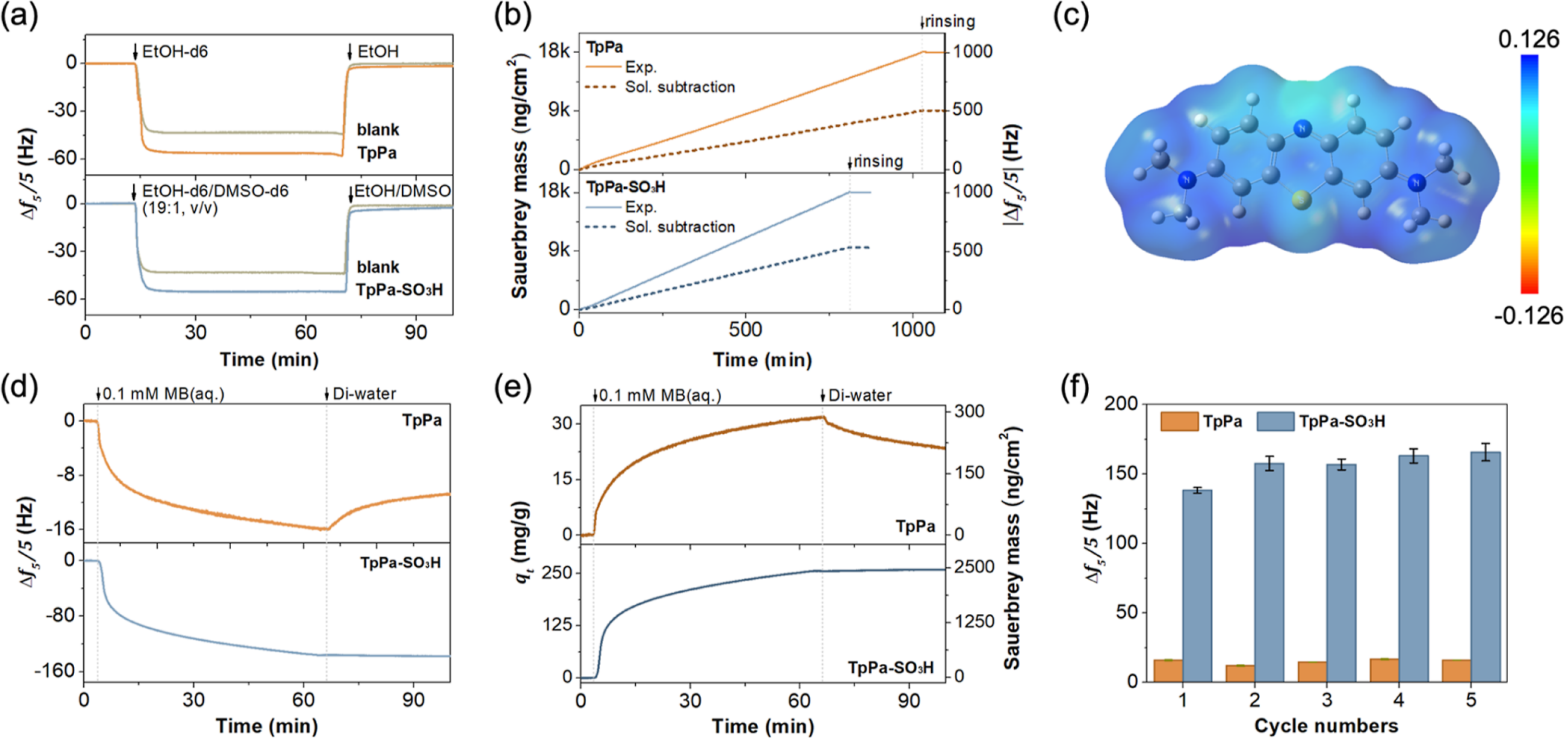
(a) QCM-D frequency responses obtained during deuterated solvent exchange experiments for blank, TpPa, and TpPa-SO_3_H-coated sensors. (b) Sauerbrey mass profiles during COF film growth, with the solid lines indicating the as-measured mass and the broken lines indicating the dry mass of the COF films. (c) Electrostatic potential of methylene blue (MB) calculated via density functional theory at the B3LYP/6-31G­(d) level. (d) Real-time frequency shifts for MB adsorption into TpPa and TpPa-SO_3_H films and (e) the corresponding adsorption quantities. (f) Reproducibility of the three independently prepared COF-coated sensors over five cycles of MB uptake and 0.1 M HCl­(aq.) treatment for COF emptying.

The frequency responses corresponding to the COF film growth process were then quantitatively analyzed to determine the deposited film mass on the sensor surface. The Sauerbrey equation (eq 3) can be applied when the viscoelastic contribution is negligible, which requires the ratio of $\left({\Delta D}_{n}\right)/\left(\frac{{\Delta f}_{n}}{n}\right)$ to be lower than 4 × 10^–7^ Hz^–1^ for a 5 MHz quartz crystal.^42^ In this work, the energy dissipation during film growth was as low as ∼1.5 × 10^–6^ (Figure 2a,b), corresponding to a ratio of ∼1.5 × 10^–9^ Hz^–1^, which is well below the threshold value for the Sauerbrey equation to be valid. The low Δ*D*_*n*_ values further confirmed that the deposited COF films were sufficiently rigid and strongly adhered to the sensor surface. Hence, the films could oscillate in sync with the quartz substrate. Since the criteria were satisfied, the measured frequency shifts ( $\frac{{\Delta f}_{5}}{5}$ ; Figure 2a,b) were converted into mass per unit area values and the resulting profiles were shown as solid lines in Figure 4b. Film growth was terminated once the frequency shift reached −1000 Hz, corresponding to a deposited mass of ∼18,000 ng·cm^–2^ for both COF films. After the solvent contributions were subtracted, the dry mass of the −1000 Hz COF films corresponded to frequency shifts of −503 Hz (∼9055 ng·cm^–2^) for TpPa (orange broken line in Figure 4b) and −530 Hz (∼9542 ng·cm^–2^) for TpPa-SO_3_H (blue broken line, Figure 4b), yielding net growth rates of dry COFs of ∼8.7 and ∼11.7 ng·cm^–2^·min^–1^, respectively.

### Adsorption and Desorption Behaviors of Methylene Blue

To evaluate the potential of using COF-coated QCM-D sensors to study the adsorption process of COFs, MB, a widely studied cationic dye in the field of environmental wastewater treatment, was selected as a model adsorbate. The molecular electrostatic potential of MB, which was calculated using Gaussian 16 version A.03 at the B3LYP/6-31G­(d) level, revealed a largely positive charge distribution (Figure 4c). Upon injection of a 0.1 mM MB solution into the flow module for 60 min, the TpPa-SO_3_H-coated sensor exhibited a significant frequency shift of −137 Hz, whereas the TpPa-coated sensor showed only a −16 Hz shift (Figure 4d). When the sample was subsequently flushed with deionized water, the TpPa-SO_3_H signal remained unchanged, indicating the strong binding of MB with the COF, whereas TpPa exhibited a positive frequency shift, suggesting MB desorption. During MB uptake for both COFs, a minimal shift in energy dissipation was observed (Figure S1), confirming that the resulting MB-COF layer was sufficiently rigid and that the Sauerbrey model (eq 3) could be applied. The masses of MB uptake after 60 min were determined to be ∼283 ng·cm^–2^ for TpPa and ∼2425 ng·cm^–2^ for TpPa-SO_3_H. On the basis of the determined COF film mass, the estimated adsorption capacities (eq 7) were ∼31 mg·g^–1^ for TpPa and ∼259 mg·g^–1^ for TpPa-SO_3_H (Figure 4e). This notable difference can be attributed to the strong electrostatic interactions between the deprotonated sulfonic anions on TpPa-SO_3_H and the cationic MB molecules.

Reusability is essential for practical sensor applications. However, despite the high adsorption capacity, simple rinsing with deionized water was insufficient to fully remove MB to regenerate empty COFs for reuse, particularly for TpPa-SO_3_H. Since the TpPa framework was reported to retain its structural stability in 9 N HCl­(aq.) for 7 days,^37^ acid treatment was employed as a regeneration strategy. Considering a substantially milder regeneration condition, the framework would retain its structural integrity. Therefore, the COF-coated sensors were immersed in 0.1 M HCl­(aq.) for 24 h. For TpPa-SO_3_H, protonation of the sulfonic groups at low pH reduced their negative charge, whereas for TpPa, acidic exposure resulted in a positive surface charge (Figure 3c). In both cases, weakened electrostatic attraction with positively charged MB molecules facilitated their desorption and restored the adsorption capacity of the COF. As shown in Figure 4f, the average adsorption capacity across three independent COF-coated sensors demonstrated that the adsorption–desorption cycles were repeatable for five rounds without performance loss, which confirms the reproducibility of the sensors and their reusability. In contrast, MB adsorption into TpPa-SO_3_H increased after the second cycle, suggesting that the initial entrance of MB molecules, although not detected by the change in energy dissipation in QCM-D, it may induce a slight structural alteration to the COF framework and allow more MB molecules to enter during subsequent rounds.

### Adsorption Kinetics and Mechanisms of Guest Molecules from Aqueous Solutions into COFs

To investigate the kinetics and mechanisms underlying the adsorption of MB into TpPa and TpPa-SO_3_H COFs, real-time QCM-D adsorption curves of samples obtained upon exposure to 0.1 mM MB solutions were analyzed. Commonly used PFO (eq 8) and PSO (eq 9) adsorption kinetics models were first employed to evaluate the experimental results. The PFO model, which was first proposed by Lagergren to describe liquid-phase adsorption, is based on the assumption that the rate-limiting step is diffusion from the bulk solution toward the solid.^46^ In contrast, the PSO model entails the assumption that the rate is limited by chemisorption. The integrated solutions of these models, which describe the adsorbed quantity (*q*_*t*_) as a function of time (*t*), are as follows^47^

8 $$q_{t.PFO}=q_{e}\left(1-e^{-k_{1}t}\right)$$

9 $$q_{t.PSO}=\frac{{q_{e}}^{2}k_{2}t}{1+q_{e}k_{2}t}$$

where *q*_e_ denotes the adsorption capacity at equilibrium (mg·g^–1^), and *q*_*t*_ is the adsorption capacity at time *t*. Moreover, *k*_1_ and *k*_2_ are the rate constants for the PFO (min^–1^) and PSO (g·mg^–1^·min^–1^) models, respectively.

As shown in Figure 5a,d, fitting the entire 60 min adsorption process with either the PFO or PSO model yielded low *R*_adj_^2^ values (Tables 1 and 2). These results indicated that the adsorption process was not governed by a single mechanism but may instead constitute a multistage process. The time-dependent fitting results provided additional insights supporting this assumption. When fitting the initial adsorption phase ranged from 0–20 min and 0–10 min, the *R*_adj_^2^ values increased substantially, especially for the first 0–10 min. The PSO model provided a better fit (*R*_adj_^2^ = 0.990 for TpPa and 0.995 for TpPa-SO_3_H) than the PFO model did (*R*_adj_^2^ = 0.980 for TpPa and 0.975 for TpPa-SO_3_H). Hence, the PSO model could be adopted to describe the initial stage of the adsorption process.

**Figure 5:**
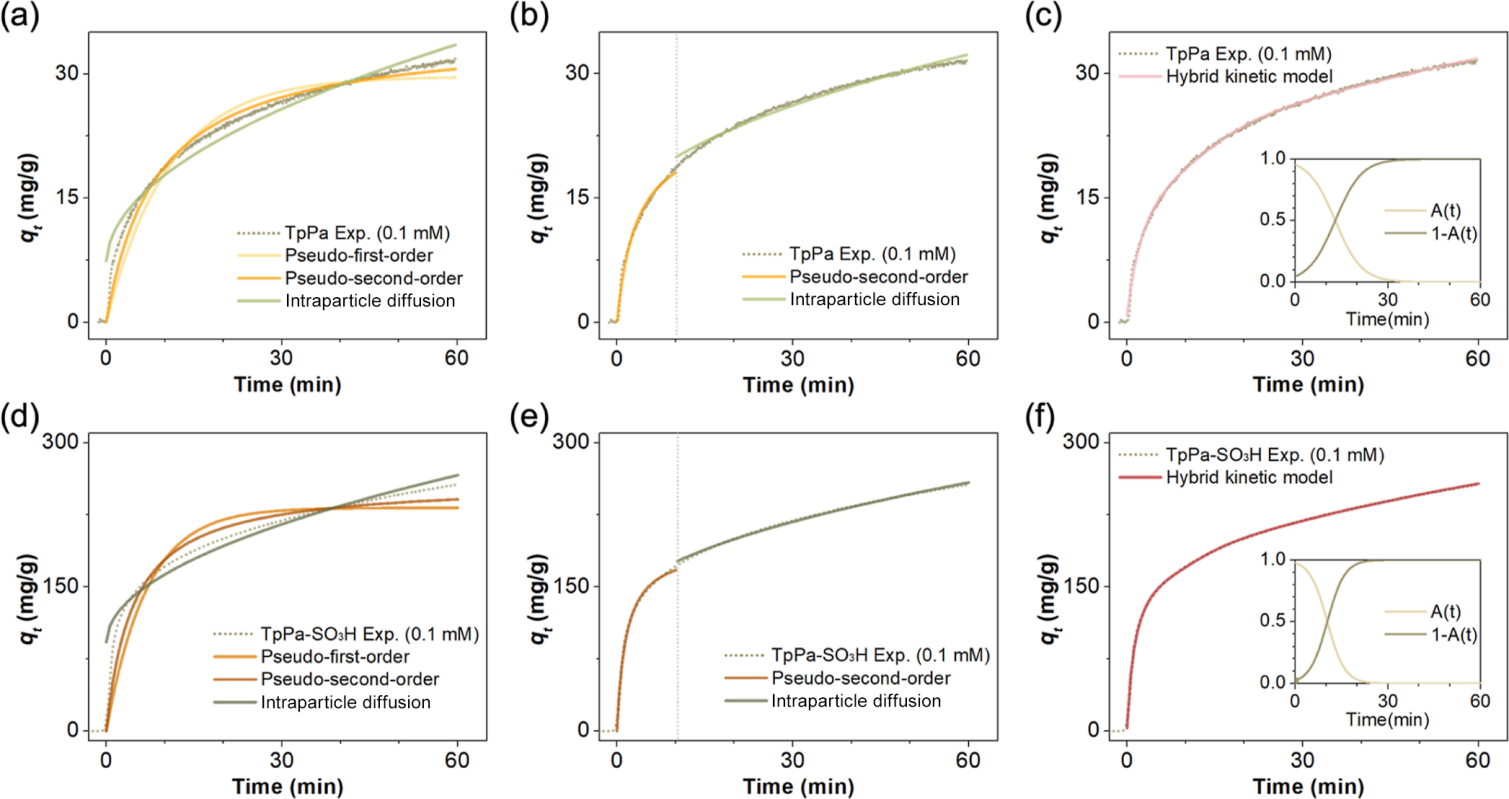
Kinetic modeling of methylene blue adsorption into TpPa and TpPa-SO_3_H films from 0.1 mM aqueous solutions. (a,d) Fitting over the full experimental period of approximately 1650 data points using the PFO, PSO, and IPD models for TpPa and TpPa-SO_3_H. (b,e) Two-stage fitting divided at 10 min with the PSO (0–10 min) and IPD (10–60 min) models for TpPa and TpPa-SO_3_H. (c,f) Fitting with the hybrid kinetic model incorporating a sigmoidal time-dependent weighting function *A*(*t*). The insets show the evolution of *A*(*t*) (contribution of the PSO mechanism) and 1 *A*(*t*) (contribution of the IPD mechanism) over time.

**Table 1:** Kinetic Parameters Obtained from the Different Models Applied to Methylene Blue Adsorption into TpPa Over Various Fitting Durations

pseudo-first order	*k*_1_ (10^–1^ min^–1^)	*q*_*e*_ (mg·g^–1^)	*R*_adj_^2^
0–60 min	0.936 ± 0.008	29.6 ± 0.1	0.941
0–20 min	1.94 ± 0.03	22.8 ± 0.1	0.970
0–10 min	3.15 ± 0.05	18.5 ± 0.1	0.980

pseudo-second order	*k*_2_ (10^–3^ g·mg^–1^·min^–1^)	*q*_e_ (mg·g^–1^)	*R*_adj_^2^
0–60 min	3.32 ± 0.03	35.0 ± 0.1	0.982
0–20 min	7.09 ± 0.12	28.5 ± 0.1	0.989
0–10 min	12.2 ± 0.3	24.2 ± 0.2	0.990

intraparticle diffusion	*k*_ip_ (mg·g^–1^·min^–0.5^)	*C*_ip_ (mg·g^–1^)	*R*_adj_^2^
60 min	3.44 ± 0.02	6.90 ± 0.10	0.954
10–60 min	2.71 ± 0.01	11.3 ± 0.05	0.987

**Table 2:** Kinetic Parameters Obtained from the Different Models Applied to Methylene Blue Adsorption into TpPa-SO_3_H Over Various Fitting Durations

pseudo-first order	*k*_1_ (10^–1^ min^–1^)	*q*_e_ (mg·g^–1^)	*R*_adj_^2^
0–60 min	1.46 ± 0.02	232 ± 0.6	0.821
0–20 min	3.58 ± 0.06	185 ± 0.6	0.928
0–10 min	5.50 ± 0.08	162 ± 0.6	0.975

pseudo-second order	*k*_2_ (10^–3^ g·mg^–1^·min^–1^)	*q*_e_ (mg·g^–1^)	*R*_adj_^2^
0–60 min	0.844 ± 0.013	259 ± 0.6	0.937
0–20 min	2.26 ± 0.03	212 ± 0.6	0.983
0–10 min	3.33 ± 0.04	193 ± 0.5	0.995

intraparticle diffusion	*k*_ip_ (mg·g^–1^·min^–0.5^)	*C*_ip_ (mg·g^–1^)	*R*_adj_^2^
60 min	22.6 ± 0.1	91.4 ± 0.8	0.937
10–60 min	18.1 ± 0.03	119 ± 0.2	0.997

To elucidate the underlying mechanism, the IPD model, which was initially developed by Weber and Morris to describe solid–liquid adsorption kinetics limited by diffusion within a solid,^48^ was chosen to account for the diffusion of MB within the pores of the COF. The solution of the IPD model can be expressed as follows

10 $$q_{t.IPD}=k_{ip}\sqrt{t}+C_{ip}$$

where *k*_ip_ is the IPD coefficient (mg·g^–1^·min^–0.5^), and *C*_ip_ is the IPD constant (mg·g^–1^).

While the IPD model alone could not describe the complete process (Figure 5a,d), it effectively complemented the PSO model. As shown in Figure 5b,e, the initial phase (0–10 min) was well described by the PSO model, whereas the later phase (10–60 min) can be described by the IPD model, with *R*_adj_^2^ = 0.987 for TpPa and *R*_adj_^2^ = 0.997 for TpPa-SO_3_H. This observation also suggested that the PSO mechanism dominated at the initial surface adsorption stage, whereas the later internal diffusion stage can be described by the IPD mechanism.

Given that a multistage adsorption process occurred, a single model was insufficient to fit all the time-dependent experimental data points as a whole. This observation is consistent with the work of Haerifar and Azizian, who also reported that kinetics can be jointly governed by surface reactions and diffusion steps.^49^ In parallel, Zhang et al. introduced a sigmoid-based adsorption model, revealing that the monotonic increase and saturation behavior of the sigmoid function rendered it well suited to describe adsorption curves involving multiple mechanisms.^50^ Inspired by these insights and mathematical descriptions, the sigmoid-like function was employed as a time-dependent weighting factor to describe the transition between the PSO- and IPD-dominated stages

11 $$q_{t}=A\left(t\right)\cdot q_{t.PSO}\left(t\right)+\left(1-A\left(t\right)\right)\cdot q_{t.IPD}\left(t\right)$$

where *A*(*t*) denotes the sigmoidal time-dependent contribution of the PSO mechanism and can be expressed as follows^50^

12 $$A\left(t\right)=\frac{1}{1+e^{n\left(t-t_{c}\right)}}$$

where *t*_c_ is the characteristic transition time when the contribution of the PSO mechanism decreases to 50%, and *n* is a steepness parameter that governs the sharpness of the transition between the two mechanisms. This empirical approach facilitated the identification of when and how rapidly the adsorption mechanism shifted between the PSO- and IPD-dominated regimes and encompassed a sigmoid model with embedded mechanistic meaning, thereby providing a rational framework for analyzing multistage adsorption kinetics.

As shown in Figure 5c,f, this hybrid kinetic model achieved an excellent fit for both COFs (*R*_adj_^2^ = 0.999 with approximately 1650 data points). The fitting results of the hybrid model (Tables 3 and 4) provided more in-depth insights into the distinct adsorption behaviors of the two COFs. Notably, TpPa exhibited a higher PSO rate constant (*k*_2_), indicating more rapid surface binding at the initial stage. Conversely, TpPa-SO_3_H demonstrated a trade-off between thermodynamics and kinetics. While its high electrostatic attraction yielded a greater equilibrium capacity (*q*_e_), the initial surface adsorption was restricted by a substantial desolvation barrier, as tightly bound counterions and water molecules must be removed from the –SO_3_H sites before effective binding of MB could occur. Thus, adsorption on TpPa-SO_3_H was thermodynamically favorable but kinetically restricted at the early stage. Once the dye molecules entered the pores, the trend reversed. TpPa-SO_3_H exhibited an IPD coefficient (*k*_ip_) nearly an order of magnitude larger than that of TpPa, originating from two synergistic effects. First, the hydrophilic –SO_3_H groups enhanced film wetting and created a water-rich environment that reduced the diffusion resistance of MB. In this regard, water contact angle measurements (Figure S2) confirmed the higher wettability of the TpPa-SO_3_H film (33°) in comparison to the TpPa film (75°). Second, although the pore channels in TpPa-SO_3_H were less well-defined than those in TpPa because of its lower degree of crystallinity (Figure 3d,e), the sulfonic acid groups dispersed throughout the disordered framework still provided adsorption sites within the solid phase, which promoted further diffusion of cationic dye molecules. In contrast, MB diffusion in TpPa proceeded mainly via slow, random movement governed by the concentration gradient.

**Table 3:** Fitted Parameters from the Hybrid Kinetic Model Describing Methylene Blue Adsorption into TpPa at Varying Concentrations

hybrid model	0.01 mM	0.05 mM	0.1 mM	1 mM
*k*_2_ (10^–3^g·mg^–1^·min^–1^)	26.5 ± 0.4	15.1 ± 0.1	12.0 ± 0.2	9.66 ± 0.09
*q*_e_ (mg·g^–1^)	10.2 ± 0.06	23.0 ± 0.04	23.1 ± 0.1	35.6 ± 0.1
*k*_ip_ (mg·g^–1^·min^–0.5^)	0.711 ± 0.005	1.68 ± 0.005	2.25 ± 0.01	2.71 ± 0.01
*C*_ip_ (mg·g^–1^)	6.07 ± 0.04	15.1 ± 0.03	14.4 ± 0.1	25.4 ± 0.1
*t*_c_ (min)	16.6 ± 0.4	14.4 ± 0.1	12.9 ± 0.2	12.2 ± 0.1
*n*	0.357 ± 0.034	0.282 ± 0.005	0.233 ± 0.005	0.191 ± 0.002
*R*_adj_^2^	0.996	0.999	0.999	0.999

**Table 4:** Fitted Parameters from the Hybrid Kinetic Model Describing Methylene Blue Adsorption Into TpPa-SO_3_H at Varying Concentrations

hybrid model	0.01 mM	0.05 mM	0.1 mM	1 mM
*k*_2_ (10^–3^g·mg^–1^·min^–1^)	0.641 ± 0.002	2.24 ± 0.01	3.55 ± 0.03	4.93 ± 0.07
*q*_e_ (mg·g^–1^)	132 ± 0.2	163 ± 0.3	187 ± 0.3	186 ± 0.6
*k*_ip_ (mg·g^–1^·min^–0.5^)	10.6 ± 0.03	14.4 ± 0.03	17.1 ± 0.04	17.4 ± 0.05
*C*_ip_ (mg·g^–1^)	38.9 ± 0.3	92.1 ± 0.2	125 ± 0.2	158 ± 0.3
*t*_c_ (min)	29.5 ± 0.3	15.1 ± 0.1	10.3 ± 0.1	7.54 ± 0.08
*n*	0.247 ± 0.013	0.363 ± 0.011	0.356 ± 0.008	0.346 ± 0.005
*R*_adj_^2^	0.999	0.999	0.999	0.999

The transition between these two mechanisms can be described by the characteristic transition time (*t*_c_) and the steepness parameter (*n*) in the time-dependent sigmoidal function. Compared with that of the TpPa film (12.9 min), the *t*_*c*_ value of the TpPa-SO_3_H film is lower (10.3 min), indicating that TpPa-SO_3_H entered the diffusion-controlled stage earlier. The sharper transition observed for TpPa-SO_3_H (*n* = 0.356 compared with 0.233 for TpPa) also suggested a more rapid shift from surface-controlled absorption to diffusion-controlled uptake.

### Effect of the Guest Concentration on Adsorption Kinetics

The influence of the initial MB concentration on the adsorption kinetics further revealed distinct behaviors for TpPa and TpPa-SO_3_H (Figure 6, Tables 3 and 4). As described by Bullen et al.,^51^ the PSO rate constant (*k*_2_) notably depends on the initial adsorbate concentration (*C*_o_) and is often inversely related to *C*_o_. This phenomenon is an intrinsic mathematical artifact of the formulation of the PSO model. Because the model does not explicitly include *C*_o_, the rate constant *k*_2_ must decrease with increasing *C*_o_ value to compensate for the corresponding increase in the equilibrium capacity (*q*_e_). This parameter dependency was considered to interpret the observed results.

**Figure 6:**
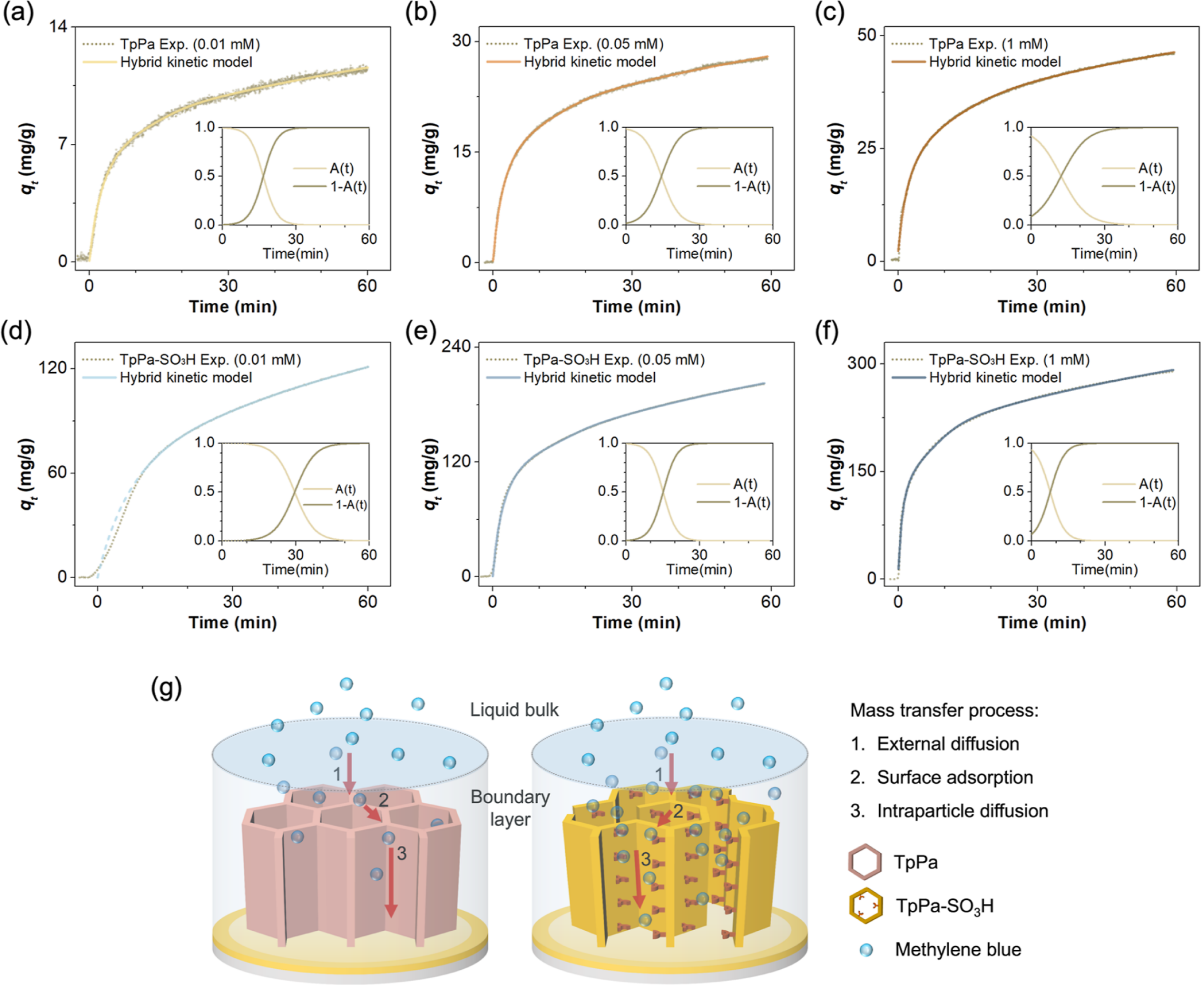
Hybrid kinetic modeling of methylene blue adsorption into TpPa and TpPa-SO_3_H films at various concentrations. (a–c) correspond to concentrations of 0.01, 0.05, and 1 mM, respectively, for the TpPa film. (d–f) show the results for the TpPa-SO_3_H film at the above-mentioned concentrations. The insets show the time-dependent weighting function, *A*(*t*) (contribution of the PSO mechanism), and its complement, 1 *A*(*t*) (contribution of the IPD mechanism), over time. (g) Schematic illustration of the adsorption processes for both COFs.

For TpPa, the decrease in *k*_2_ from 2.65 × 10^–2^ to 9.66 × 10^–3^ g·mg^–1^·min^–1^ as *C*_o_ increased from 0.01 to 1 mM conformed with the expected mathematical dependency described above. The transition time (*t*_c_) from surface adsorption to IPD decreased from 16.6 to 12.2 min, suggesting more rapid saturation of surface sites due to the higher concentration of guest molecules in the environment and an earlier onset of IPD-controlled uptake.

In contrast, TpPa-SO_3_H demonstrated the opposite trend, with *k*_2_ increasing from 6.41 × 10^–4^ to 4.93 × 10^–3^ g·mg^–1^·min^–1^ with increasing *C*_o_. This behavior suggested that the introduction of sulfonic acid groups altered the inherent mathematical inverse relationship of the PSO model between *k*_2_ and *C*_o_. This change could be explained by considering that the strong electrostatic attraction between the MB molecules and the –SO_3_H groups facilitated the surface uptake process, thereby providing a kinetic enhancement that was sufficient to numerically outweigh the parameter dependency of the model. This enhanced uptake process, especially at a lower concentration of 0.01 mM (a more limited MB supply), also caused poor agreement between the PSO model and the experimental data (Figure 6d). To better visualize this deviation, the linear form of the PSO plot in Figure S3 was employed, revealing a significant departure from the linear model during the first 9.8 min of the process. This finding suggested that the adsorption process was initially constrained by the supply of MB rather than surface reactions. Under such low-concentration conditions, the system experienced a supply limited period, leading to a transient depletion of the adsorbate near the surface. Since this phase violated the fundamental assumption of a constant concentration in the PSO model, the hybrid model was applied only to the experimental data recorded after 9.8 min, with the initial segment represented by extrapolation (indicated by the broken line in Figure 6d). In terms of the transition time (*t*_c_) from the PSO- to IPD-dominated regimes, TpPa-SO_3_H followed a concentration-dependent trend similar to that of TpPa, decreasing significantly from 29.5 to 7.54 min with increasing concentration. This pattern confirmed that greater driving forces accelerate the transition to the IPD stage with the –SO_3_H pendent group.

An analysis of the IPD stage provided further insights into the underlying mass transfer mechanism. Unlike the different trends observed with *k*_2_, the IPD coefficient (*k*_ip_) consistently increased with increasing concentration for both TpPa and TpPa-SO_3_H, reflecting the strong driving force resulting from the high concentration gradients of MB within the COF. This effect was more significant in TpPa-SO_3_H because of the electrostatic facilitation between the anionic framework and cationic guest, resulting in much higher *k*_ip_ values than those of TpPa. Furthermore, the value plateaued above 0.1 mM (*k*_ip_ = 17.1 mg·g^–1^·min^–0.5^), suggesting that the internal diffusion rate had reached its maximum.

In conclusion, the distinct multistage mass transfer processes of guest molecules in the two COFs revealed by the hybrid model are shown in Figure 6g. The process begins with the transport of MB molecules from the liquid bulk across the boundary layer to the COF surface (1). In the functionalized TpPa-SO_3_H framework, although surface adsorption (2) is initially restricted by the desolvation of –SO_3_H sites, these groups provide a notable driving force for cationic molecules and yield *q*_*e*_ values superior to those of nonfunctionalized TpPa. Once the molecules enter the pores, the –SO_3_H sites significantly facilitate mass transport, driving a much higher IPD flux (3) than that in the nonfunctionalized TpPa. Consequently, the incorporation of sulfonic acid groups facilitated more efficient progression of guest molecule transport, effectively overcoming the slower and concentration-gradient-dependent diffusion observed in the TpPa framework.

## Conclusions

In this work, a QCM-D-based methodology that facilitates quantitative analysis of multistage adsorption kinetics in β-ketoenamine-linked 2D COF thin films (TpPa and TpPa-SO_3_H) in aqueous media was proposed. By integrating the flow-based *in situ* synthesis with high temporal resolution QCM-D monitoring, homogeneous, rigid, and adherent films with controllable growth were obtained. Through deuterated solvent exchange experiments, the intrinsic mass of the COF films was determined, allowing quantitative evaluation of adsorption capacities. Utilizing MB as a model adsorbate, TpPa-SO_3_H demonstrated superior adsorption capacity compared to TpPa due to the electrostatic attraction between the cationic MB and the sulfonic acid groups. It was found that single mechanism could not describe the entire adsorption process. Therefore, a hybrid model with a sigmoidal time-dependent weighting function was proposed to capture the two-stage adsorption kinetics. The first stage corresponds to surface adsorption controlled PSO kinetics, whereas the later stage is dominated by IPD. A comparison of the adsorption kinetics between TpPa and TpPa-SO_3_H revealed that the pendent functional group governs the mechanism. While TpPa is governed by concentration-driven diffusion, the electrostatic driving force in the TpPa-SO_3_H framework promotes mass transport inside the solid. As a result, the adsorption capacity and IPD rate of TpPa-SO_3_H were nearly an order of magnitude greater than those of unfunctionalized TpPa.

Beyond the specific findings of this work, this methodology provides a platform for real-time and quantitative studies of host–guest interactions in porous materials, offering insights into multistage adsorption kinetics for advancing various applications of reticular materials.

## Supplementary Material



## Notes

Figure S1. Frequency and dissipation responses upon the introduction of a 0.1 mM methylene blue aqueous solution over 90 min (a) TpPa film. (b) TpPa-SO_3_H film. Figure S2. Water contact angle of (a) TpPa film and (b) TpPa-SO_3_H film. Figure S3. Linear plots of the pseudo-second-order model for MB adsorption into TpPa-SO_3_H at 0.01 mM (PDF)
