# Supplementary material for: Flow-Based In Situ Synthesis of Covalent Organic Framework Thin Films and Liquid-Phase Quartz Crystal Microbalance with Dissipation Monitoring (QCM-D) Analysis of Adsorption Kinetics
Source: ACS Appl Mater Interfaces. 2026 Jun 1;18(22):32094–106. doi: 10.1021/acsami.6c06434 (PMC13266702; doi:10.1021/acsami.6c06434)
Supplement: Supplementary file 1 [file am6c06434_si_001.pdf]

Supporting Information

Flow-Based *In Situ* Synthesis of Covalent Organic Framework Thin Films and Liquid-Phase Quartz-Crystal Microbalance with Dissipation Monitoring (QCM-D) Analysis of Adsorption Kinetics

Wen-Yi Yu[1,2], Pei-Chen Huang[1,2], Yun-Wen You[2], I-Chia Huang[2], Cheng-Si Tsao[1,4], and Jing-Jong Shyue[*1,2,3]

[1] Department of Materials Science and Engineering, National Taiwan University, Taipei 10617, Taiwan

[2] Research Center for Applied Sciences, Academia Sinica, Taipei 11529, Taiwan

[3] Program in Semiconductor Devices, Materials, and Hetero-integration, Graduate School of Advanced Technology, National Taiwan University, Taipei 10617, Taiwan

[4] National Synchrotron Radiation Research Center, Hsinchu 30076, Taiwan

*To whom correspondence should be addressed. Telephone +886(2)2787-3137. Fax +886 (2)2787-3122. E-mail: shyue@gate.sinica.edu.tw. ORCID: 0000-0002-8508-659X.

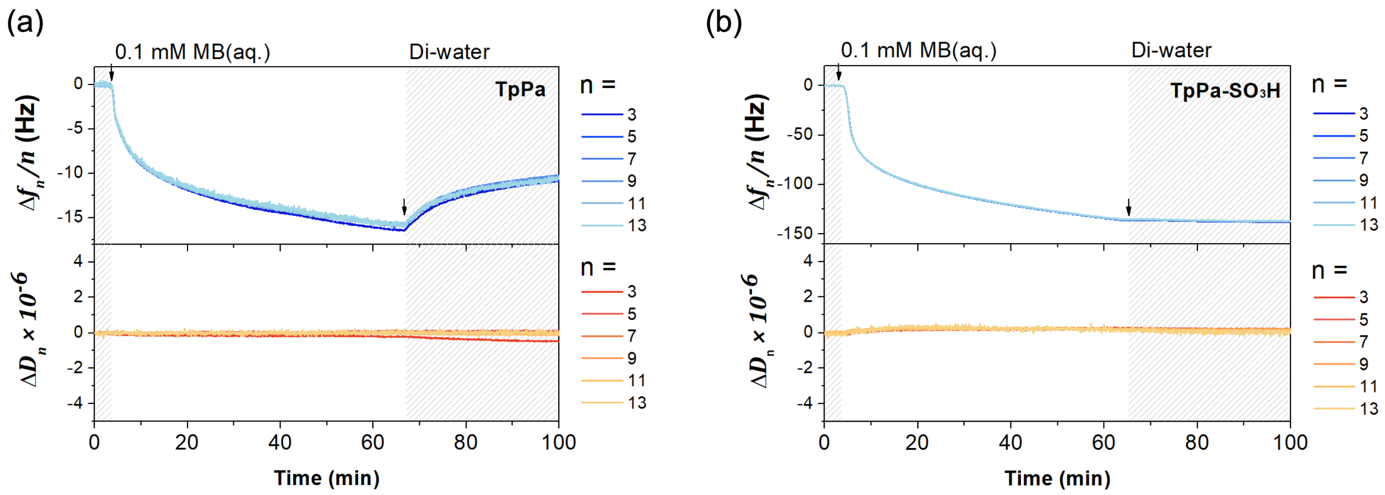

Figure S1. Frequency and dissipation responses upon the introduction of a 0.1 mM methylene blue aqueous solution over 90 minutes. **(a)** TpPa film. **(b)** TpPa-SO_3H film.

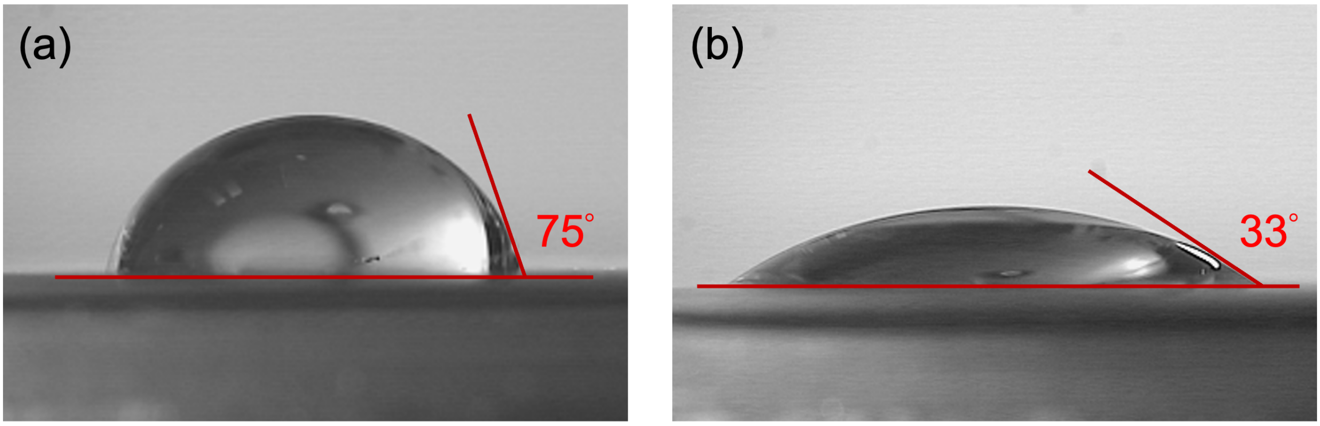

Figure S2. Water contact angle of **(a)** TpPa film and **(b)** TpPa-SO_3H film.

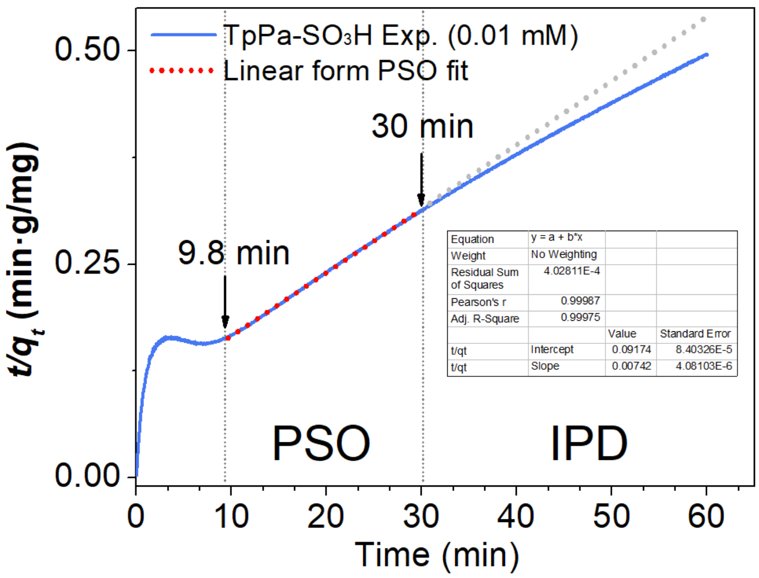

Figure S3. Linear plots of the pseudo-second-order model for MB adsorption into TpPa-SO_3H at 0.01 mM.
